# The therapeutic potential of red beetroot (*Beta vulgaris* L.) intake on muscle atrophy in immobilized mouse skeletal muscles

**DOI:** 10.1002/fsn3.4335

**Published:** 2024-09-23

**Authors:** Seyedeh Elnaz Nazari, Nima Khalili‐Tanha, Fatemeh Babaei, Ali Jafarzadeh Esfahani, Ghazaleh Khalili‐Tanha, Fereshteh Asgharzadeh, Fatemeh Khojasteh‐Leylakoohi, Seyyedeh Zahra Asghari, Mousa‐Al‐Reza Hadjzadeh, Seyed Mahdi Hassanian, Gordon Ferns, Amir Avan, Reza Rezvani, Majid Khazaei

**Affiliations:** ^1^ Department of Medical Physiology, Faculty of Medicine Mashhad University of Medical Sciences Mashhad Iran; ^2^ Metabolic Syndrome Research Center Mashhad University of Medical Sciences Mashhad Iran; ^3^ Basic Sciences Research Institute Mashhad University of Medical Sciences Mashhad Iran; ^4^ Student Research Committee Mashhad University of Medical Sciences Mashhad Iran; ^5^ Department of Nutrition, Faculty of Medicine Mashhad University of Medical Sciences Mashhad Iran; ^6^ Department of Medical Education Brighton & Sussex Medical School Brighton UK; ^7^ Medical Genetics Research Center Mashhad University of Medical Sciences Mashhad Iran

**Keywords:** beetroot, *Beta vulgaris* L., limb immobilization, muscle atrophy, nitrite, oxidative stress

## Abstract

Skeletal muscle atrophy is the reduction in muscle mass and function caused by an imbalance in protein synthesis and degradation. Inflammation has been shown to accelerate protein degradation during periods of muscle inactivity. We investigated the potential therapeutic effects of beetroot extract (BRE) in reducing inflammation and oxidative stress to prevent muscular atrophy after a short period of immobilization. We divided 36 male BALB/c mice into three groups: control, muscular atrophy mice, and muscular atrophy mice treated with BRE (*n* = 12). Each group was further divided into two subgroups: (1) 7‐day immobilization and (2) 10‐day recovery. BRE was administered orally at a dose of 300 mg/kg for 17 days. We assessed the anti‐inflammatory and antioxidative effects of BRE using ELISA and RT‐PCR assays. Hematoxylin–eosin staining was used to measure the cross‐sectional area (CSA) of muscle fibers, and grip strength tests were performed to assess muscle strength. BRE administration increased muscle weight, CSA, and grip strength in mice with immobilization‐induced muscle atrophy. It also suppressed inflammation and oxidative stress biomarkers in atrophic muscle fibers. Higher nitrate levels and lower Troponin I (TnI) concentrations were observed in the BRE‐treated group, indicating improved muscle function and structure. These findings suggest that BRE may have therapeutic benefits in improving muscle mass and function and warrant further studies in humans, particularly in individuals with low physical activity levels.

## Graphical abstract

Skeletal muscle atrophy is a big concern for athletes after muscle injuries. The recovery from many injuries sustained in athletic training or competition often requires an extensive period of muscle disuse and immobilization. Muscle atrophy is considered to be reduced muscle mass and function, and it occurs when the balance of protein synthesis/degradation is disturbed. In this study, we examined the therapeutic potential of beetroot extract (BRE) in modifying inflammation and oxidative stress to decrease muscular atrophy after a short period of immobilization.

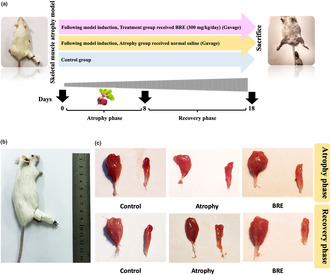

## 1 INTRODUCTION

Skeletal muscles are important plastic and dynamic tissues that play essential roles in force generation and movement. They contain approximately 50%–70% of all body proteins. Generally, muscle mass and strength are influenced by the balance between protein synthesis and protein degradation in the muscle cell population (Kubat et al., 2023; Park et al., 2013). Some physical and pathological conditions, like denervation, microgravity, immobilization, chronic diseases, and malnutrition, can affect muscle mass and function. Due to the adaptive nature of the muscle tissue, it responds to these conditions through different phenomena, including changes in capillary distribution, muscle fiber size, myosin isoforms, extracellular connective tissue constituents, and cytoplasmic organelles. All of these conditions can cause skeletal muscle atrophy (Dumitru et al., 2018; Matarneh et al., 2021). Muscle atrophy can be limited to an individual or a group of different muscles (e.g., in patients who are unable to walk or move after orthopedic surgeries) (Bowerman et al., 2018; Sedano et al., 2013). The underlying mechanism behind muscle atrophy is not well established; however, some molecular pathways are considered to influence the onset of the muscle atrophy process. It has been shown that following immobilization, pro‐inflammatory cytokine expression is increased in skeletal muscle cells (Parker et al., 2022). Increased pro‐inflammatory cytokines can help to disrupt protein synthesis and degradation balance in muscle fibers (Agrawal et al., 2023). Interleukin‐6 (IL‐6) is one of these pro‐inflammatory biomarkers that has a dual function; it acts both as a muscle growth inhibitor and as a pro‐inflammatory cytokine (Yakabe et al., 2018). The other cytokines, which have been studied more extensively in muscle atrophy, are tumor necrosis factor‐alpha (TNF‐α) and Interleukin‐1 (IL‐1). It was demonstrated that high levels of pro‐inflammatory cytokines, such as IL‐6 and TNF‐α, are more expressed in elderly patients following physical inactivity (Alghadir et al., 2021). Troponin I, as a troponin complex component, also has a regulatory function in skeletal and cardiac muscles (Yamada et al., 2020). As demonstrated earlier, microgravity can cause muscle atrophy in which mRNA levels of troponin I have been reduced in the muscles during this condition (Jiang et al., 2023). It was also reported that prolonged immobilization of muscle tissues increases the production of reactive oxygen species (ROS) that can result in muscle fiber injury (Silva et al., 2022). Increased ROS levels could help muscle atrophy by affecting related signaling pathways (Hussain et al., 2010; Powers, 2014). Skeletal muscle atrophy is also associated with the patient's morbidity and mortality. There are just a few therapeutic approaches to preventing skeletal muscle atrophy. Therefore, introducing new products is essential to improving these problems.

Nowadays, natural products, as potential modern medicines, are used to treat various pathological conditions (Jones, 2014; Saha et al., 2019). Red beetroot (Beta vulgaris L.) has attracted extensive attention in the past few years due to its bioactive components, such as ascorbic acid, carotenoids, phenolic acids, nitrate, and flavonoids (Ceclu & Nistor, 2020). Various studies have shown that red beetroot inhibits inflammation and oxidative damage by reducing gene expression of related factors (Albasher et al., 2020; Al‐Harbi et al., 2021; Lechner & Stoner, 2019). In this study, we investigated the therapeutic potential of RBE in the prevention of skeletal muscle atrophy through anti‐inflammatory and antioxidant activity.

## 2 MATERIALS AND METHODS

### 2.1 Preparation of BRE

The process of producing lyophilized alcohol extracts from beetroot involves multiple intricate steps that are crucial to ensuring the efficient extraction and preservation of bioactive compounds (Babagil et al., 2018). Initially, fresh beetroot samples that were purchased from a local store were meticulously cleaned, peeled, and diced into small pieces to increase the surface area for extraction. Subsequently, the beetroot pieces were immersed in a solution containing ethanol (20%) and water to facilitate the extraction of compounds. To enhance the efficiency of the extraction process, the mixture was allowed to macerate for a period of 3 days. Following this, the liquid extracts underwent filtration to eliminate any solid plant material, resulting in a clarified solution rich in extracted compounds.

Further concentration of the extracts was achieved using a rotary evaporator, which served to decrease the volume and amplify the concentration of the desired compounds. Subsequent freezing of the concentrated extracts preceded the final step of lyophilization, involving the removal of moisture content through sublimation under reduced pressure and controlled heat. This delicate process ensures the preservation of the structural integrity and chemical composition of the compounds present in the extract.

The resulting beetroot powder obtained post‐lyophilization is a finely textured product containing crucial bioactive compounds such as inorganic nitrate, phenolics, ascorbic acid, carotenoids, and betalains. In a specialized analysis undertaken as part of our study, the nitrate content of the beetroot powder was quantified to be 9.8 mg per gram of powder (using the spectrophotometric method), emphasizing the significance of this method in retaining the bioactive properties of the beetroot extract. As documented, the preparation of lyophilized beetroot extracts encompasses a series of intricate steps that are pivotal in ensuring the retention of bioactive compounds in the final product.

### 2.2 Animals and chemicals

BALB/c mice, aged 8–10 weeks, weighing 25–30 g, were obtained from the animal facilities of Mashhad University of Medical Sciences. The animals were maintained under standard conditions of temperature (23 ± 25°C), light (12 h/12 h light–dark cycles), and relative humidity (70%). In this study, all animal experiments conformed to the ethical guidelines accepted by Mashhad University of Medical Sciences (MUMS) (ethical approval code: 4000877). All reagents for malonyl dialdehyde (MDA) were purchased from Sigma Chemical Co (St. Louis, MO, USA). A nitrate/nitrite assay kit was provided by Bio‐techne Co (MPLS, MN, USA), and ELISA kits for IL‐6, TNF‐α, and troponin I (TnI) were obtained from eBioscience Co (San Diego, CA, USA). The RNA isolation kit and cDNA synthesis kit were both purchased from Qiagen Inc. (Hilden, DEU).

**FIGURE 1:**
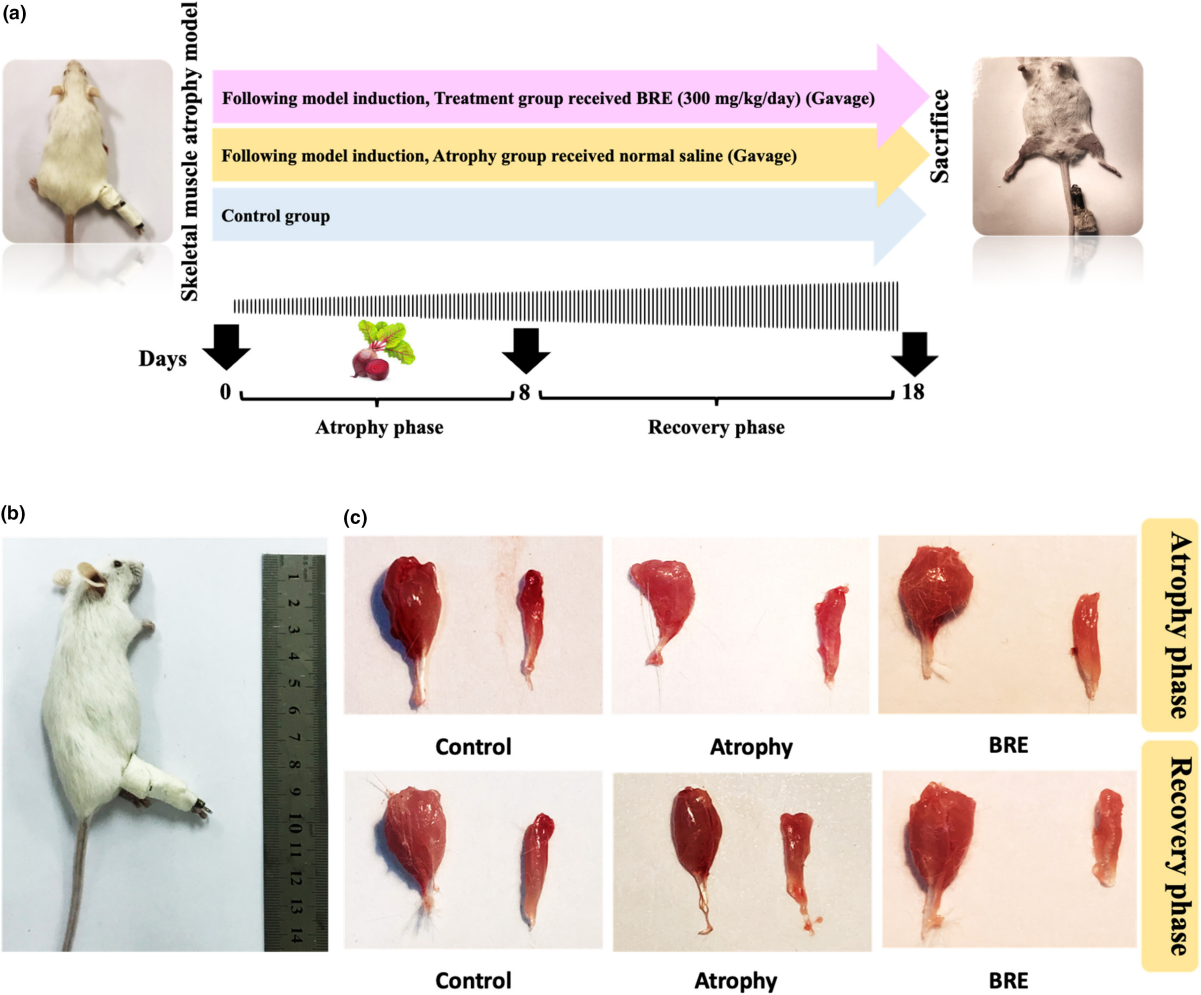
BRE treatment decreased disuse‐induced muscle atrophy. (a) A schematic representation of the study protocol. (b) Unilateral hindlimb immobilization to reproduce a disuse muscle atrophy model. (c) Macroscopic demonstration of muscle atrophy in different experimental groups. In two phases of atrophy and recovery, the gastrocnemius and soleus muscle mass were significantly higher in the BRE‐treated group than in the atrophy group.

Thirty‐six mice were divided equally into three groups (*n* = 12): Group 1: Control; Group 2: Muscular atrophy mice; Group 3: Muscular atrophy mice administered BRE. Mice assigned to the control group did not receive any intervention. The animals assigned to the treatment group received 1 daily oral infusion with 300 mg/kg of BRE dissolved in water for 17 days (Raish et al., 2019) (Figure 1a).

### 2.3 Experimentally induced muscle atrophy

As previously described, unilateral hindlimb immobilization was used to reproduce a disuse muscle atrophy model (Guitart et al., 2018). Briefly, the right hindlimb was shaved and enveloped using surgical tape. A 1.5‐microcentrifuge tube was applied to maintain the foot in a plantar‐flexed position to obtain the maximal atrophy of the gastrocnemius and soleus muscles (Figure 1b). The experimental procedure was performed without general anesthesia using a restraining device. As the tube weight was approximately 0.5 g, it did not affect the usual mobility of the animals. After induction of muscular atrophy, each group was subdivided into two groups: (1) 7‐day immobilization and (2) 10‐day recovery groups. The splint was removed in the recovery phase to let the mice move freely in order to investigate muscle recovery. At the end of the experiment, mice from all principal groups were sacrificed. In each mouse, general anesthesia was performed intraperitoneally with a 70 mg/kg ketamine and 10 mg/kg xylazine mixture. For macroscopic evaluations of muscular atrophy, the gastrocnemius and soleus muscles were collected and weighted in all cases at the time of sacrifice. Tissue samples were stored in 10% formalin and liquid nitrogen for histological and biochemical analysis.

### 2.4 Limb grip strength test

Grip strength was assessed on Days 1, 7, and 17 during the study period using a grip strength measurement device (DS2‐110500 N, Toyohashi, Japan) (Figure 3a). Mice were placed on a grid connected to a grip strength dynamometer. After the animals held the grid, they were pulled horizontally to measure the maximum force of grip. An average of three measurements was used for the analysis.

### 2.5 Histological analysis

Muscle tissue samples were fixed in neutral‐buffered formalin (10%), dehydrated, and embedded in paraffin. Samples were sliced into 4 μm‐thick sections using a microtome (Shandon, Pittsburgh, PA 15275, CA, USA) and stained with hematoxylin and eosin (H&E) to evaluate the muscle cross‐section area (CSA). NIH imaging software (Image J) was used to outline and measure the muscle tissue CSA. Myofiber images were obtained using standard light microscopy (Carl Zeiss Meditec, Berlin, Germany) with a medium magnification (20×).

### 2.6 Biochemical assessment

Tissue samples were removed, homogenized in PBS (phosphate‐buffered saline), and centrifuged at 3000 rpm for 5 min; then the supernatant was collected to measure tissue concentrations of IL‐6, TNF‐α, and TnI by mouse ELISA (ZellBio GmbH, Ulm, Germany) and nitrite level (Griess reagent system, Promega, USA), according to the manufacturer's instructions. For the evaluation of MDA levels, both serum and tissue supernatant were applied to the TBA‐reactive compounds. The absorption of the liquid mixture was read at 435 nm, and the MDA concentration was assessed by C (M) = A/1.65 × 105.

### 2.7 Extraction of RNA and quantitative real‐time PCR

Trizol reagent was used to isolate muscle total RNA by following the manufacturer's protocol (Parstoos, Mashhad, Iran). The quantity of isolated RNA was determined using a NanoDrop detector (NanoDrop Technology‐1000, USA). A reverse transcription reaction was performed using a cDNA synthesis kit protocol (Cina Colon, Tehran, Iran). qRT‐PCR was conducted using a light cycler (Roche) and SYBER Green qPCR Master mix to perform all the PCR reactions. GAPDH was used to normalize the mRNA levels of targeted genes, and a comparative method (2^−∆Ct^) was applied to quantify relative expression ratios.

### 2.8 Statistical analysis

All data were presented as mean ± standard deviation (SD) and analyzed by the one‐way ANOVA test. The Shapiro–Wilk normality test was used to assess the data normality. A rate of *p* < .05 was considered statistically significant.

## 3 RESULTS

### 3.1 The effect of BRE on muscle mass, grip strength, and CSA of muscle fibers

**FIGURE 2:**
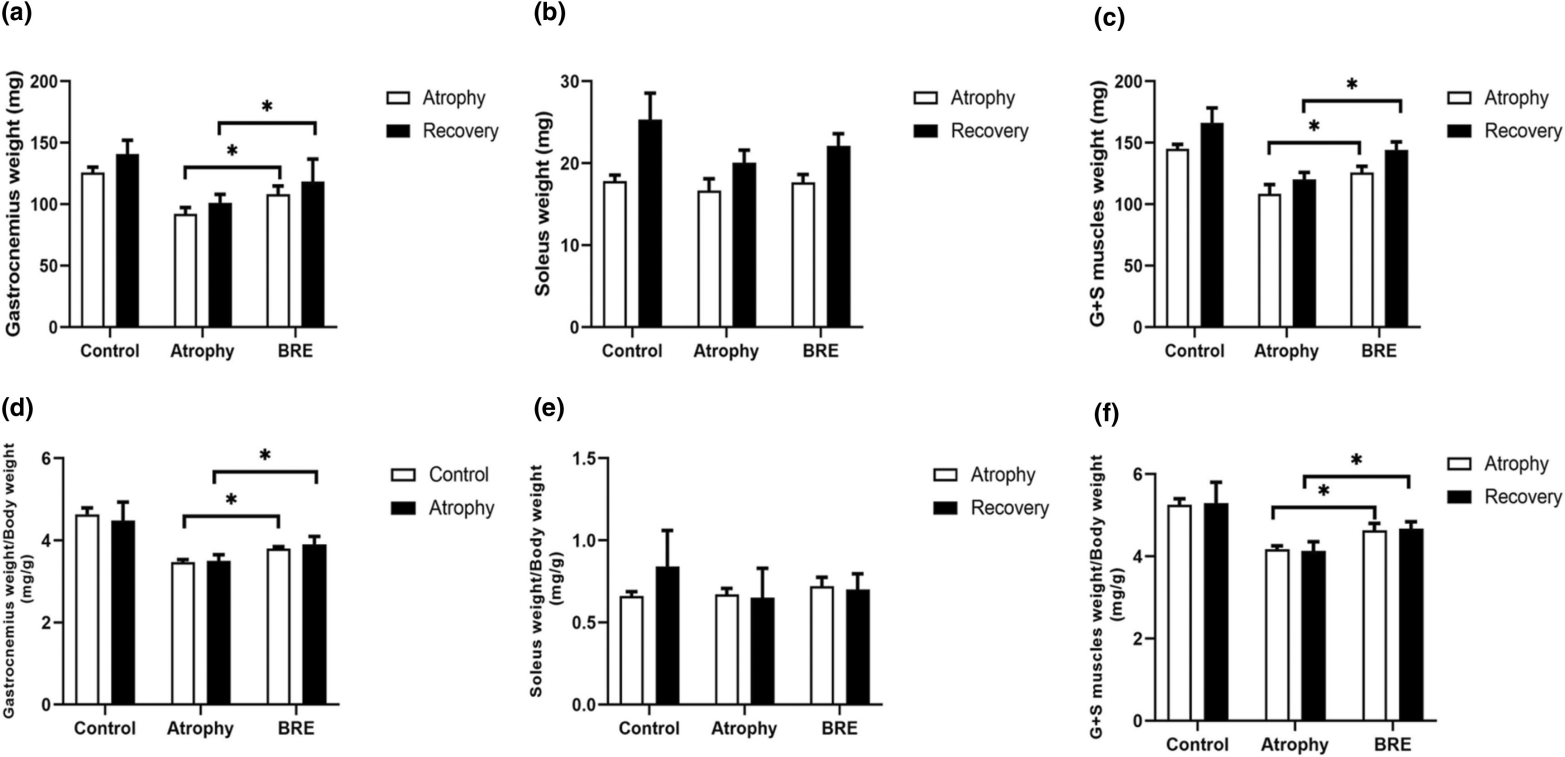
BRE treatment increased hindlimb skeletal muscle weight. (a–c) Fresh weights of gastrocnemius and soleus muscles in all groups at the end of each phase. (d–f) Body‐mass normalized muscle weights of gastrocnemius and soleus muscles. Data are presented as mean ± SD (*n* = 12) (*p < .05).

As described in the methods, the wet weight of all gastrocnemius and soleus muscles was measured in both phases of immobilization and recovery. The gastrocnemius muscle weight in the BRE‐treated group was significantly increased compared to the atrophy group (*p < .05). The soleus muscle weight did not show a significant difference in comparison to the atrophy group. The total muscle weight (Gastrocnemius + Soleus) was significantly higher in the BRE‐treated group (Figure 1c) (Figure 2a–f) (*p < .05).

**FIGURE 3:**
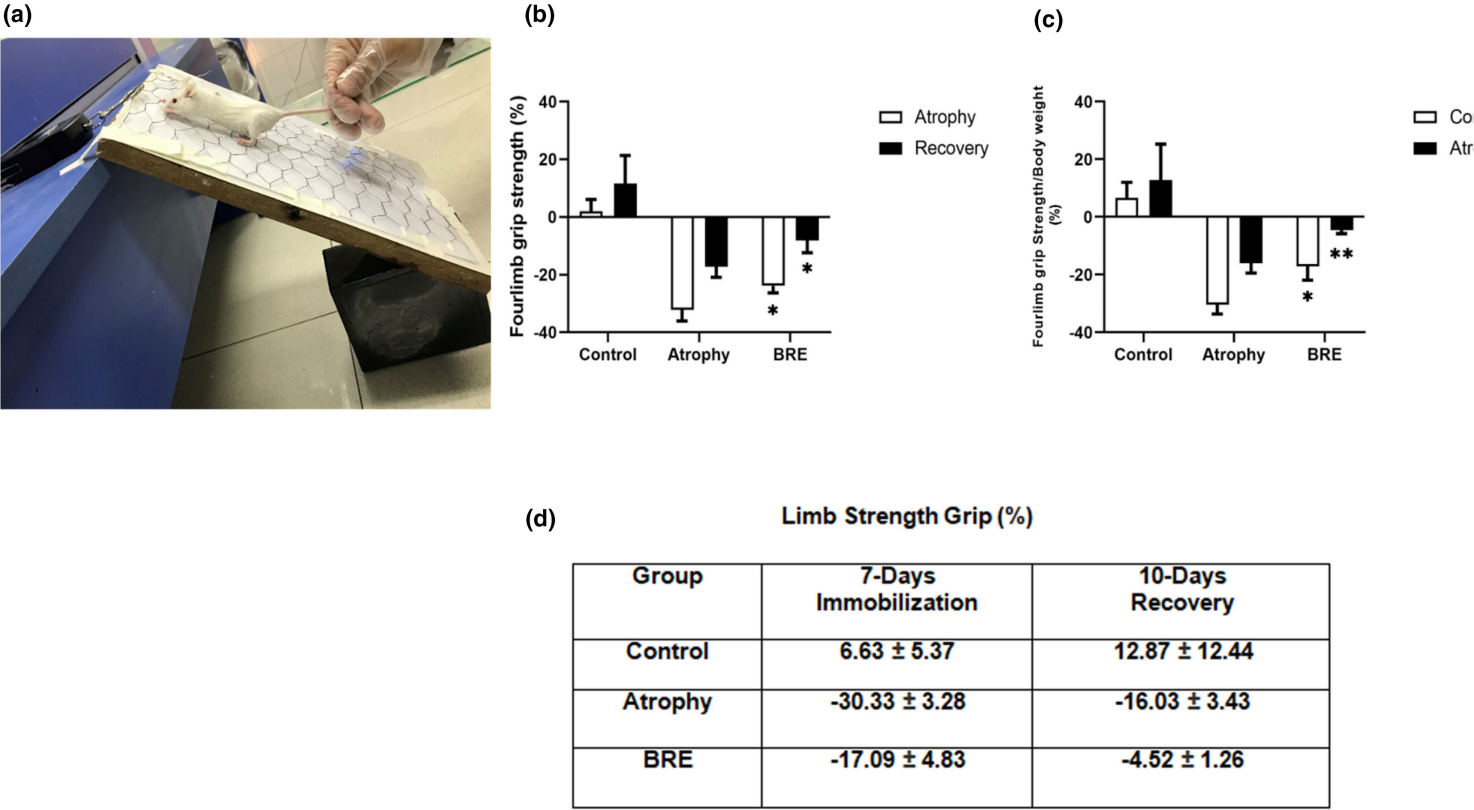
BRE treatment improved hindlimb muscle strength in atrophic BALB/c mice. (a) The instrument used to assess grip strength in the mice forelimbs. (b) The grip strength of the forelimbs was measured at three time points (on Days 1, 7, and 17) and (c, d) calculated per body weight. Data are presented as mean ± SD (*n* = 12) (**p < .01) (*p < .05).

The grip strength test was used to evaluate muscle strength capacity during both phases of immobilization and recovery. At the end of each period, the forelimb grip strength was decreased by (−30.33% ± 3.28%) (−16.03% ± 3.43%) in the atrophy group and significantly increased by approximately (−17.09% ± 4.83%) (−4.52% ± 1.26%) in the BRE‐treated group (**p < .01) (*p < .05). Figure 3b–d shows a detailed quantitative grip strength percentage for each group in two phases of immobilization and recovery.

**FIGURE 4:**
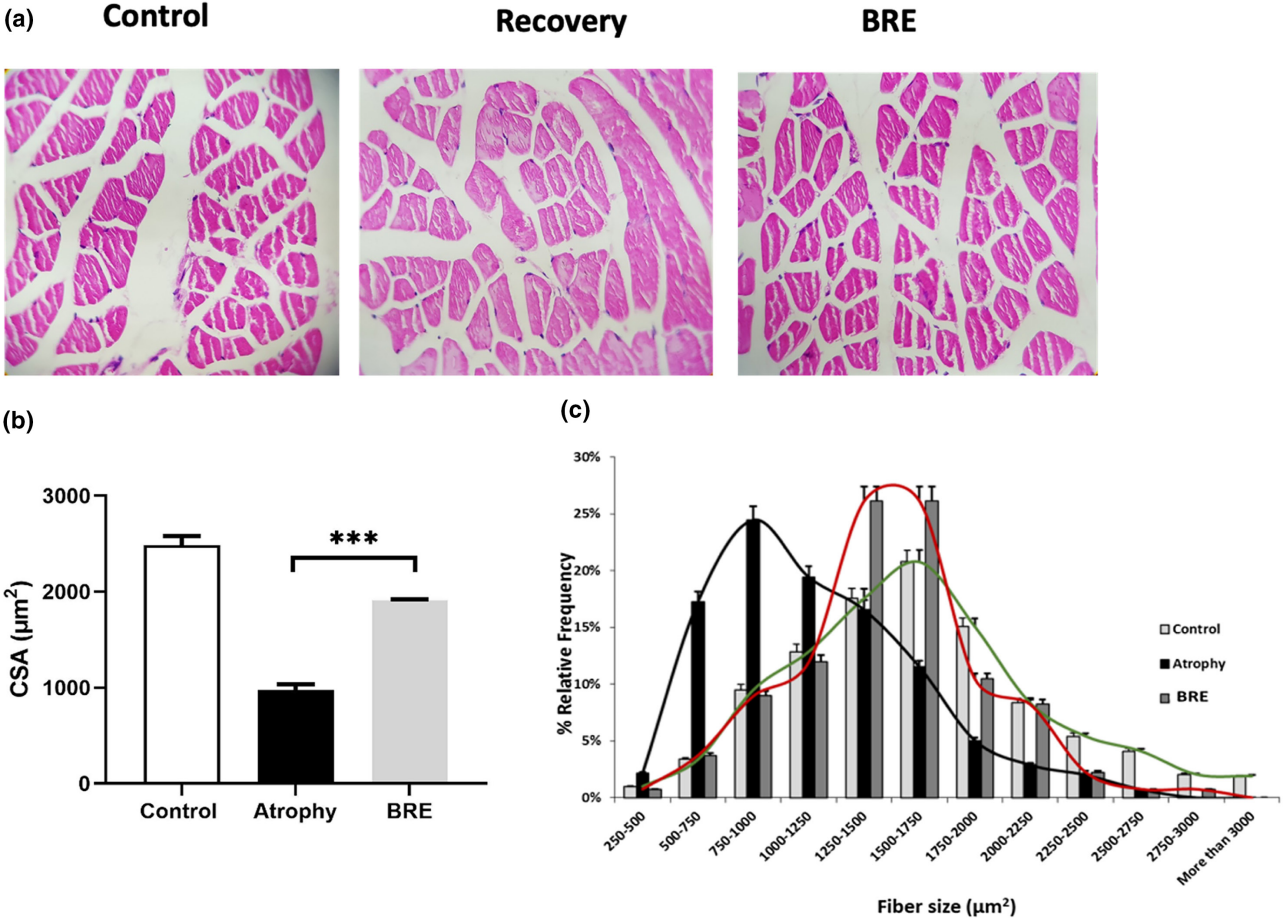
BRE treatment improved fiber cross‐sectional area (CSA) after the atrophy period. (a) The representative microscopic H&E staining of gastrocnemius muscle tissue (20×). (b) Mean fiber CSA and (c) relative frequency distribution of muscle fiber size in gastrocnemius muscle. Data are presented as mean ± SD (*n* = 6) (***p < .001).

The histological assessment of muscle tissues showed that atrophy decreased the fiber size and cross‐sectional area of each fiber, and BRE treatment could significantly increase it at the end of the immobilization phase (***p < .001) (Figure 4a,b). The relative frequency of different muscle fiber types in the atrophy group was between 500 and 1250 μm^2^; BRE could positively affect the fiber size distribution toward the larger sizes. The greater frequency of muscle fibers was between 1250 and 1750 μm^2^ in the treatment group (Figure 4c).

### 3.2 The effect of BRE on inflammation, oxidative stress, nitrite/nitrate content, and Tn I concentration

**FIGURE 5:**
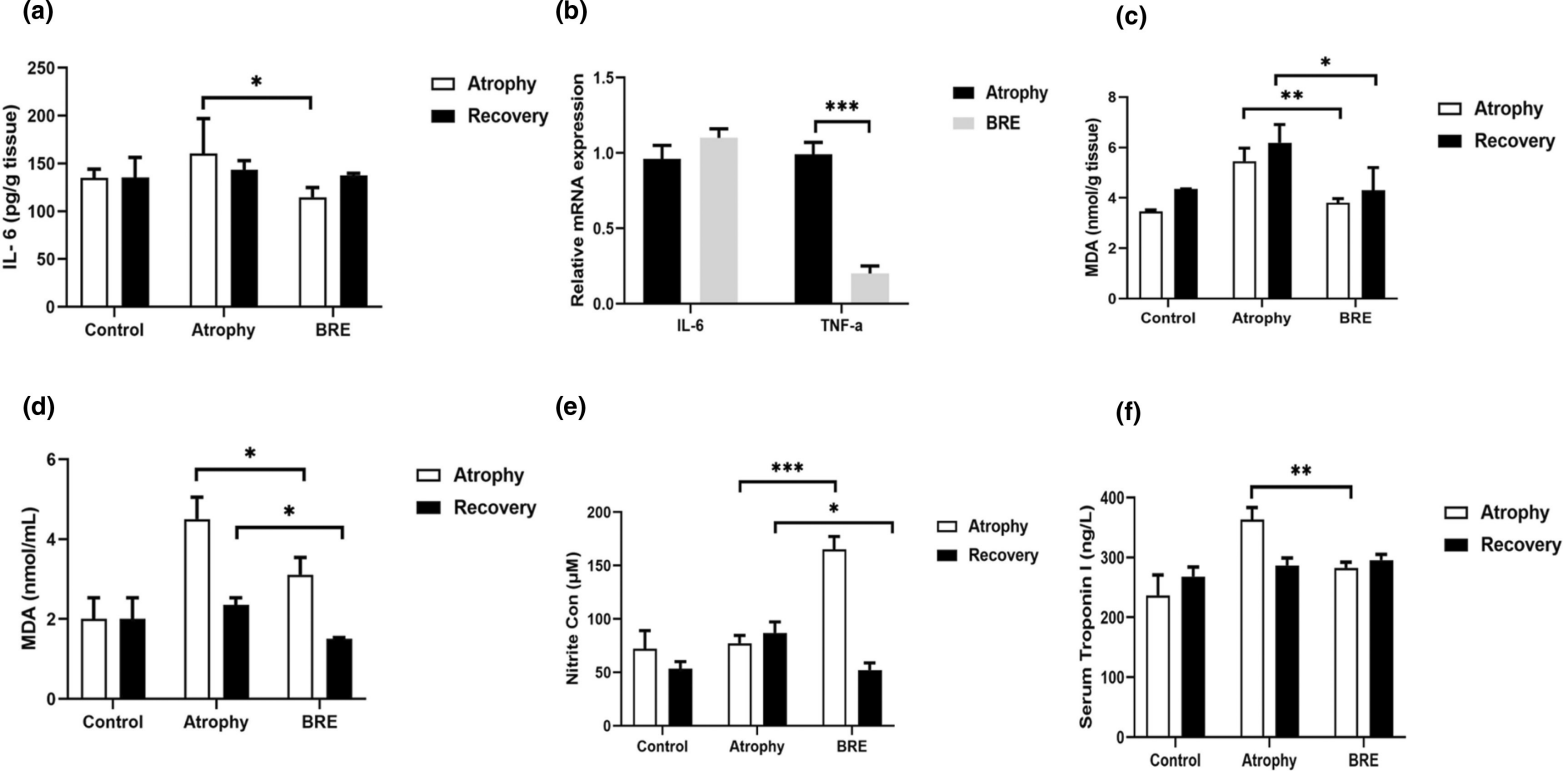
BRE treatment attenuated skeletal muscle atrophy factors in mice. (a, b) The mRNA and protein levels of IL‐6 and TNF‐α in all gastrocnemius muscle tissues. (c, d) The tissue and serum concentrations of MDA, nitrite, and Tn I in each experimental group (e, f). Data are presented as mean ± SD (*n* = 12) (***p < .001; **p < .01; and *p < .05).

Our results showed that BRE could reduce the recruitment of pro‐inflammatory cytokines in the atrophic muscles after the immobilization period. In the BRE‐treated group, the concentration of IL‐6 was significantly reduced in comparison to the atrophy group (*p < .05) (Figure 5a). Furthermore, the mRNA expression level of TNF‐α was significantly decreased in the BRE group than in the atrophy group (**p < .01) (Figure 5b).

The tissue and serum MDA concentration, as an important lipid peroxidation product, has enhanced after 7 days of immobilization. After both phases of immobilization and recovery, the MDA levels in the BRE‐treated group displayed a significant decrease in comparison with the atrophy group (* *p* < .05) (** *p* < .01) (Figure 5c,d).

Our finding showed that the nitrite content of the BRE group differed significantly in both phases. The BRE group had a significantly higher nitrite content than the atrophy group (*p < .05) (***p < .001) (Figure 5e).

As demonstrated in Figure 5, serum troponin‐I concentrations in atrophic animals showed a significant reduction in the phase of immobilization. Treatment with BRE could significantly increase serum Tn I levels in comparison with the atrophy group (**p < .001) (Figure 5f).

## 4 DISCUSSION

Skeletal muscle atrophy, a loss of muscle mass and function, occurs when the balance of protein synthesis and degradation is disturbed. In this experiment, we demonstrated the potential value of BRE in the improvement of skeletal muscle atrophy in a murine model. Our finding showed that BRE could significantly decrease inflammatory responses and suppress oxidative stress in atrophic muscles, causing an improvement in muscle strength and function. This study supports the therapeutic potential of BRE in preventing muscle atrophy.

Various studies have shown that the microenvironment of skeletal muscle fibers is altered during inflammatory conditions (Chazaud et al., 2009; Pizza et al., 2005; Tidball, 2005). A variety of signaling pathways, destroying protein synthesis and degradation balance, can be activated by different pro‐inflammatory cytokines (Bowen et al., 2015). Various studies have shown that beetroot, as a source of important phytochemicals, has high anti‐inflammatory and antioxidant capabilities (Esatbeyoglu et al., 2014; Pietrzkowski et al., 2010; Vidal et al., 2014; Weitzberg & Lundberg, 2013). In a study, Clifford et al. (2016) demonstrated that short‐term administration of beetroot juice alleviated muscle soreness and decrement by suppressing leukocyte activation and secretion of pro‐inflammatory cytokines. In another study, Kozłowska et al. (2020) showed that beetroot juice supplementation can reduce muscle damage, oxidative stress, and inflammation in trained athletes. The anti‐inflammatory potential of BRE was also evaluated by Jein et al. In vivo experiments revealed that BRE downregulated NF‐Kappa B‐related gene expression in aged kidneys (Jain et al., 2011). Similarly, in our study, BRE decreased inflammatory responses by reducing the mRNA and protein concentrations of inflammatory mediators in a murine model of skeletal atrophy.

Similar to the anti‐inflammatory properties of BRE, various studies are showing that oxidative stress in muscles could help with muscle wasting and weakness (Berretta et al., 2020; Chen et al., 2022; Moylan & Reid, 2007). BRE is considered a strong antioxidant defense to protect cells from oxidative stress (Raish et al., 2019). Gamal et al. have shown that rats administered BRE had notably decreased inflammation and oxidative stress during kidney dysfunction (El Gamal et al., 2014). Esatbeyoglu et al. have also demonstrated that in diabetic rats, BRE could decrease renal fibrosis by suppressing oxidative stress biomarkers (Esatbeyoglu et al., 2014). Moreover, the antioxidant activity of red beetroot was confirmed by the study conducted by Kujawska et al. In this study, beetroot juice administration protects animals from oxidative stress by reducing lipid peroxidation formed by liver injury (Kujawska et al., 2009). Beetroot powder has also demonstrated antioxidant potential in hyperuricemia‐induced rats by preventing MDA production significantly (Wulandari et al., 2021). In line with these data, our result suggests that BRE has an effective regulatory role on the oxidant/antioxidant balance during the muscle atrophy process.

BRE contains a high inorganic nitrate (NO3^−^) concentration, and its intake has been confirmed effective in enhancing blood nitric oxide (NO) (Domínguez et al., 2018). Given the role of NO in improving blood flow and vasodilation with a beneficial effect on muscular activity, several studies have shown the positive effect of BRE supplementation in the improvement of muscular power and hypertrophy (Gliemann et al., 2019; Pereira‐Neto et al., 2021; Van Hoorebeke, 2015). Ferguson et al. have confirmed the beneficial effect of beetroot juice (BRJ) on the regulation of skeletal muscle perfusion. They concluded that NO3^−^ supplementation elevates muscle blood volume and metabolic performance during BRJ consumption (Ferguson et al., 2020). In a study, Kokkinoplitis et al. demonstrated that a higher dose of BRJ positively affects muscular performance and strength in different healthy subjects (Kokkinoplitis & Chester, 2014). Poredos et al. have also shown the potential effect of an acute BRJ diet on men's muscle strength and endurance (Poredoš et al., 2022). Consistent with these data, our study indicated that BRE, as a NO3‐rich dietary supplement, could improve muscle function and structure during muscle atrophy.

## 5 CONCLUSIONS

Based on our data, it was observed that beetroot extracts potentially possess therapeutic and preventive effects in improving muscle atrophy. These findings support the need for further investigation through clinical trials to evaluate the effectiveness of emerging therapeutic strategies for treating this condition.

## AUTHOR CONTRIBUTIONS

**Seyedeh Elnaz Nazari:** Investigation (lead); writing – original draft (lead). **Nima Khalili‐Tanha:** Investigation (equal); writing – original draft (equal). **Fatemeh Babaei:** Investigation (equal); software (equal); validation (equal). **Ali Jafarzadeh Esfahani:** Writing – original draft (equal); writing – review and editing (equal). **Ghazaleh Khalili‐Tanha:** Investigation (equal); methodology (equal); writing – original draft (equal). **Fereshteh Asgharzadeh:** Investigation (equal); visualization (equal); writing – original draft (equal). **Fatemeh Khojasteh‐Leylakoohi:** Methodology (equal); writing – original draft (equal). **Seyyedeh Zahra Asghari:** Investigation (equal); methodology (equal). **Mousa‐Al‐Reza Hadjzadeh:** Writing – review and editing (equal). **Seyed Mahdi Hassanian:** Writing – original draft (equal). **Gordon Ferns:** Writing – review and editing (equal). **Amir Avan:** Supervision (equal); writing – review and editing (equal). **Reza Rezvani:** Methodology (equal); supervision (equal); writing – review and editing (equal). **Majid Khazaei:** Investigation (equal); software (equal); supervision (equal); writing – review and editing (equal).

## FUNDING INFORMATION

This research was supported by the Elite Researcher Grant Committee under award number 4002436 from the National Institute for Medical Research Development (NIMAD).

## CONFLICT OF INTEREST STATEMENT

The authors have no conflicts of interest to declare.

## ETHICS APPROVAL

This experiment was performed following the guidelines authorized by the Research Ethics Committee of Mashhad University of Medical Sciences with the ID Number “IR.MUMS.MEDICAL.REC.4000877.”

## CONSENT TO PARTICIPATE

The final manuscript has been approved by all co‐authors.

## Data Availability

The data for this study are available on request from the corresponding authors.
