# Correction to “The Therapeutic Potential of Red Beetroot (*Beta vulgaris* L.) Intake on Muscle Atrophy in Immobilized Mouse Skeletal Muscles”

**DOI:** 10.1002/fsn3.70679

**Published:** 2025-09-01

Nazari, S. E., N. Khalili‐Tanha, F. Babaei, A. Jafarzadeh Esfahani, G. Khalili‐Tanha, F. Asgharzadeh, F. Khojasteh‐Leylakoohi, S. Z. Asghari, M. A. Hadjzadeh, S. M. Hassanian, G. Ferns, A. Avan, R. Rezvani, and M. Khazaei. “The Therapeutic Potential of Red Beetroot (*Beta vulgaris* L.) Intake on Muscle Atrophy in Immobilized Mouse Skeletal Muscles.” *Food Science & Nutrition* 12, no. 11 (September 23, 2024): 9085–93. https://doi.org/10.1002/fsn3.4335.

In the originally published version of the article, citation and attribution of previously published data/images were inadvertently missing. The corrected text appears below along with corrections to statistical methods used in the study.

**Proper citation for previously published data/images:**

Figures 1–5 in this article overlap with figures from previously published studies by the same research group. These overlaps occur because the control and atrophy groups were shared across multiple studies conducted simultaneously in the same laboratory.

The following corrections ensure proper attribution of previously published data:

- Figures 1, 2, 3, and 5 in this article were adapted from:
  ○ Figures 1–4 in Seifi et al. (2024)
  ○ Figures 1–6 in Seifi et al. (2024)
  ○ Figures 1–5 in Nazari et al. (2023)
- Figure 4 (Cross‐Sectional Area, CSA) was adapted from:
  ○ Figure 4 in Seifi et al. (2024)
  ○ Figure 5 in Seifi et al. (2024)
  ○ Figure 4 in Nazari et al. (2023)
- Figure 4 (Histopathological Analysis) was adapted from:
  ○ Figure 5 in Seifi et al. (2024)
  ○ Figure 5 in Seifi et al. (2024)
  ○ Figure 4 in Nazari et al. (2023)

The corrected captions appear below.

**Correction of statistical errors:**

- Standard deviation (SD) was erroneously used in the text and figures instead of standard error (SE). The ANOVA with LSD post hoc analysis needs to be revised to align with best statistical practices. Given that only three groups were compared, LSD was deemed statistically appropriate. In section 2.8, “All data were presented as mean ± standard deviation (SD) and analyzed by the one‐way ANOVA test.” should be corrected to: “All data were presented as mean ± standard Error (SE) and analyzed by the one‐way ANOVA test.” The corrected figure captions appear below.

The authors apologize for these errors and confirm that these corrections do not alter the scientific conclusions of the paper.

**FIGURE 1** (a) A schematic representation of the study protocol. (b) Unilateral hindlimb immobilization to reproduce a disuse muscle atrophy model. (c) Macroscopic demonstration of muscle atrophy in different experimental groups. In two phases of atrophy and recovery, the gastrocnemius and soleus muscle mass were significantly higher in the BRE‐treated group than in the atrophy group. Adapted from Figures 1–4 in Seifi et al. (2024); Figures 1–6 in Seifi et al. (2024); and Figures 1–5 in Nazari et al. (2023).

**FIGURE 2** (a–c) Fresh weights of gastrocnemius and soleus muscles in all groups at the end of each phase. (d–f) Body‐mass normalized muscle weights of gastrocnemius and soleus muscles. Data are presented as mean ± SE (*n* = 12) (*p < 0.05). Adapted from Figures 1–4 in Seifi et al. (2024); Figures 1–6 in Seifi et al. (2024); and Figures 1–5 in Nazari et al. (2023).

**FIGURE 3** (a) The instrument used to assess grip strength in the mice forelimbs. (b) The grip strength of the forelimbs was measured at three time points (on Days 1, 7, and 17) and (c, d) calculated per body weight. Data are presented as mean ± SE (*n* = 12) (**p < 0.01) (*p < 0.05). Adapted from Figures 1–4 in Seifi et al. (2024); Figures 1–6 in Seifi et al. (2024); and Figures 1–5 in Nazari et al. (2023).

**FIGURE 4** BRE treatment improved fiber cross‐sectional area (CSA) after the atrophy period. (a) The representative microscopic H&E staining of gastrocnemius muscle tissue (20×). (b) Mean fiber CSA and (c) relative frequency distribution of muscle fiber size in gastrocnemius muscleData are presented as mean ± SE (*n* = 6) (***p < 0.001). The Histopathological Analysis was adapted from Figure 5 in Seifi et al. (2024); Figure 5 in Seifi et al. (2024); and Figure 4 in Nazari et al. (2023). The CSA was adapted from Figure 4 in Seifi et al. (2024); Figure 5 in Seifi et al. (2024); and Figure 4 in Nazari et al. (2023).

**FIGURE 5** (a, b) The mRNA and protein levels of IL‐6 and TNF‐α in all gastrocnemius muscle tissues. (c, d) The tissue and serum concentrations of MDA, nitrite, and Tn I in each experimental group (e, f). Data are presented as mean ± SE (*n* = 12) (***p < 0.001; **p < 0.01; and *p < 0.05). Adapted from Figures 1–4 in Seifi et al. (2024); Figures 1–6 in Seifi et al. (2024); and Figures 1–5 in Nazari et al. (2023).
